# Risk and Protective Factors of Disordered Eating in Adolescents Based on Gender and Body Mass Index

**DOI:** 10.3390/ijerph17249238

**Published:** 2020-12-10

**Authors:** Marios Argyrides, Elly Anastasiades, Evangelia Alexiou

**Affiliations:** Eating and Appearance Research Laboratory, Neapolis University Pafos, 8042 Paphos, Cyprus; e.loizou@nup.ac.cy (E.A.); evangelia.alexiou@psychogenesis.com.cy (E.A.)

**Keywords:** feeding and eating disorders, risk factors, protective factors, adolescent, body mass index, body image

## Abstract

The current study aimed to identify potential psychosocial risk and protective factors contributing to eating disorders in adolescents, and observe any differences between genders and Body Mass Index (BMI) categories. A cross-sectional survey was carried out with a total of 2605 (1063 male) adolescents, who were assessed for disordered eating, body-image satisfaction and investment, appearance/weight-related anxiety, situational dysphoria, media influences, self-esteem, and body appreciation. The results revealed that weight/appearance-related anxiety and situational dysphoria were the most significant risk factors for both genders. Pressures from the media posed a significant risk only for males and the internalization of the thin ideal only for females; however, the internalization of the athletic ideal did not pose as a significant risk factor. Compared to gender, these risk factors did not differ based on BMI. Additionally, body appreciation was found to be a robust protective factor (unlike global self-esteem) for both genders, and across all BMI groups. The findings indicate that the most significant risk and protective factors of eating disorders do not differ largely for male and female adolescents or different BMIs. Intervention and prevention programs would therefore benefit from the inclusion of exercises that reduce the constructs of weight/appearance-related anxiety and situational dysphoria, and promote body appreciation.

## 1. Introduction

The etiology of eating disorders has proven to be complex and multifaceted, and has been found to be accounted for by a variety of factors including genes, neurocircuitry, physiology, and psychosocial factors [1]. Keel and Forney [1] highlight a number of psychosocial factors that have been found to influence eating disorders, as well as the significant influence of the internalization of the thin ideal, and of weight concerns as risk factors for eating disorders. These include personality traits such as negative emotionality [2] and perfectionism [3], as well as peer pressure and peer influence [4]. Epidemiological studies show that the peak risk periods for developing an eating disorder are during adolescence and early adulthood [5], and that the risk of onset is higher in females [1]. Cultural differences have also been found to have a significant role in the development of eating disorders through thin-idealization in females, and athletic/muscular-idealization in males [6,7]. Thin-ideal internalization in females has received much support in the development of eating disorders, and intervention programs have been developed and implemented [8]. Additionally, the muscular/athletic-ideal has also received support for the development of disordered eating in males, but research has shown this connection to be complex, and few intervention programs have been developed [9].

A further construct that has been proposed as a possible psychosocial risk factor in the onset of eating disorders is that of situational body image dysphoria [10]. This construct assesses the extent of dysphoria experienced by individuals in social settings due to their perceived body image. However, studies on the significance of situational body image dysphoria in the onset of eating disorders are rather limited. Other psychological factors have also been found to play a protective role against the onset of eating disorders. Among these are body appreciation [11] and self-compassion [12].

Eating disorders have been found to vary across cultures [13]. The island-country of Cyprus has received significant attention in the body image and eating disorder literature, [14] for the following reasons: (a) the year-round warm weather and very mild winters mean that more revealing clothing is generally worn; (b) there is great emphasis on social and physical status, resulting from the economic rise of the unoccupied part of the country after the 1974 Turkish invasion; (c) there is a significant lack of prevention programs that address body image and eating disorder issues; and (d) Cyprus has been found to have the highest levels of weight-related anxiety and investment in appearance among other European nations, factors which are well-supported risk-factors in the development of eating disorders [15]. Therefore, research on the adolescent population concerning risk and protective factors for eating disorders is deemed necessary in order to inform targeted prevention programs, which can be implemented to protect against the development of eating disorders.

Therefore, the purpose of the current study was to identify protective and risk factors for the development of eating disorders in a large sample of adolescents based on their gender and their Body Mass Index (BMI). This purpose is essential because it will allow specific populations to be targeted concerning the development and implementation of future prevention programs. The study is also significant since it attempts to provide more information about males and eating disorders, separate BMI groups, and makes use of the construct of situational body image dysphoria, which is not often used in the literature. Based on previous literature and the purpose of the study, the following research questions were assessed:

1. Are there differences in risk factors predicting disordered eating between males and females, and to what extent do body appreciation and global self-esteem serve as protective factors for both genders?
2. How do the risk factors predicting disordered eating differ between BMI categories, and to what extent do body appreciation and global self-esteem serve as protective factors for all BMI categories?

## 2. Materials and Methods

### 2.1. Ethical Considerations

The current study was carried out following the rules of the Declaration of Helsinki of 1975. Ethical approval for this study was obtained from the Centre for Educational Research and Evaluation in Cyprus. The principals of each school were informed about the aims of the study, as well as the data collection procedure, and their approval was sought prior to its commencement. Similarly, written informed-consent was given by parents of the participants, after they too were informed of the aims of the study and data collection procedures. Participation in the study was voluntary.

### 2.2. Sample

Participants consisted of a convenience sample of 2605 male and female adolescents. All public and private, urban and rural schools, across the unoccupied region of Cyprus were invited to participate in the study. The inclusion criteria were; 1, attending 9th to 12th grade; and 2, being a native Greek speaker.

### 2.3. Measures

*Disordered Eating Attitudes and Behavior.* Disordered eating attitudes and behavior were measured using the Greek version of the Eating Attitudes Test-26 (EAT-26) [16,17], which identifies the symptoms of disordered eating according to feelings, attitudes, and behaviors. The EAT-26 comprises 26 items, on three subscales; dieting, bulimia and food preoccupation, and oral control. The items are rated on a 6-point Likert-type scale, ranging from 1 = always, to 6 = never. A composite score is calculated based on the 26 items. A score of 20 or higher indicates a high level of engagement with body shape and dieting behavior, as well as a heightened risk of developing eating disorder pathology. Since the main purpose of the study was to identify individuals who were at-risk of developing eating disorders, only the composite score was used in this study. The EAT-26 has reported reliability coefficients ranging from 0.86 to 0.90. For the current study, the internal consistency reliability coefficient of the composite score was 0.87.

*Body-image satisfaction, body-image investment and appearance/weight-related anxiety*: Appearance satisfaction, investment in appearance, and appearance/weight–related anxiety were measured using the Greek version of the Multidimensional Body–Self Relations Questionnaire–Appearance Scales (MBSRQ–AS) [18,19]. Four of the five subscales were used; the 7–item appearance evaluation subscale, which measures feelings of physical attractiveness and satisfaction with appearance, the 12–item appearance orientation subscale, which measures the extent of investment in appearance, the 4–item overweight preoccupation subscale, which measures appearance/weight–related anxiety, and the 9-item body areas satisfaction scale, which measures satisfaction with specific parts of the body. All of the items were rated on a 5–point Likert–type satisfaction scale ranging from 1 = very dissatisfied to 5 = very satisfied, and 1 = strongly disagree to 5 = strongly agree. The subscales of the current measure have been found to have good psychometric properties among both genders and different cultural groups with alpha coefficients above 0.80. For the current sample, the alpha coefficient for the appearance evaluation, appearance orientation, overweight preoccupation, and body area satisfaction subscales were 0.82, 0.81, 0.86, and 0.85 respectively.

*Self-esteem*: Self-esteem was measured using the Greek version of the Rosenberg Self-Esteem Scale [20,21], which consists of 10 items that assess levels of global self–worth, based on the positive and negative beliefs and perceptions about oneself. The questionnaire uses a 4–point Likert–type scale ranging from 1 = strongly disagree, to 4 = strongly agree. Reliability coefficients for The Rosenberg Self-Esteem Scale are very high, ranging from 0.87 to 0.93. The alpha coefficient for the current sample was 0.87.

*Situational body image dysphoria*: The Greek version of the Situational Inventory of Body Image Dysphoria–Short version (SIBID-S) [22,23] was used to measure body image-related dysphoria in social situations. This measure consists of 20 items which respondents are required to rate, according to how often they experience body-image dysphoria, or distress, in each of 20 identified situations—including both social and nonsocial contexts, and activities related to exercising, grooming, eating, intimacy, physical self-focus, and appearance alterations. The items are scored on a 5-point Likert-type rating scale ranging from 0 = never to 4 = always. The composite total score has a highly robust reliability coefficient of 0.96. The alpha coefficient for the current sample was 0.94.

*Media influences*: The Greek version of the Sociocultural Attitudes Towards Appearance Questionnaire–3rd version (SATAQ–3) [24,25] was used to measure media influences. This 30-item measure consists of four subscales: internalization-general (internalization of the thin-ideal), internalization-athlete (internalization of the athletic-ideal), pressures (perceived pressures from the media regarding appearance), and information (whether the media are perceived as a good source of information regarding appearance). The items are scored on a 5–point Likert–type scale ranging from 1 = definitely disagree to 5 = definitely agree. The measure has excellent psychometric properties across several populations and ages with internal Cronbach alpha coefficients ranging from 0.84 to 0.93. For the current sample, the Cronbach’s alphas for internalization; general internalization, athlete, pressures, and information, were 0.92, 0.82, 0.94, and 0.86, respectively.

*Body appreciation*: Body appreciation was measured using the Greek version of the Body Appreciation Scale-2 (BAS-2) [26,27], which measures various aspects of positive body image; an individual’s respect for, acceptance of, and favorable opinions towards their body. This measure is comprised of 10 items that are rated on a 5-point Likert-type scale ranging from 1 = never to 5 = always. The measure has excellent psychometric properties with internal Cronbach alpha coefficients ranging from 0.86. The alpha coefficient for the current sample is 0.92.

### 2.4. Procedure

Cross-sectional data collection was carried out at the schools, during school hours by a research team. The participants completed the demographic survey which included questions on gender, age, height and weight, socioeconomic status, and geographical region of the school (urban/rural), followed by the questionnaire comprising the aforementioned measures. BMI was calculated for all participants, based on their reported height and weight using the Center for Disease Control Child and Adolescent BMI scale [28]. These were categorized as underweight, normal weight, overweight, and obese. Due to the low number of participants in the obese category, for the purposes of this study, the overweight and obese categories were combined, resulting in three final BMI categories which were used in the analysis: underweight, normal weight and overweight/obese. Total scores were calculated for each of the measures. The data were analyzed using SPSS statistical software version 25 (IBM SPSS Inc., Chicago, IL, USA). Descriptive statistical analyses and stepwise multiple regression analyses were carried out.

## 3. Results

The demographic characteristics of the sample indicated that, of the 2605 participants, 1063 were male [40.8%] and 1542 were female [59.2%]. Participants had a mean age of 15.22 years (SD = 1.23) with a range of 13 to 18 years, a mean height of 166.89 cm (SD = 8.43 cm; range 110–200 cm) and a mean weight of 58.72 kg (SD = 12.04; range 36–124 kg). The mean BMI for the full sample was 21.00 (SD = 3.55; range 12.35–49.59), with 594 participants (22.8%) in the underweight category, 1722 (66.1%) in the normal weight category, 227 (8.7%) in the overweight category, and 62 (2.4%) in the obese category. Eighty-seven (3.5%) participants had low socioeconomic status, 321 (12.8%) had middle-low socioeconomic status, 1412 (56.2%) had middle socioeconomic status; 569 (22.7%) had middle-high socioeconomic status and 122 (4.9%) had high socioeconomic status, with the majority of the participants coming from middle class families. The majority of the participants attended urban (2048; 78.6%) schools and 557 participants (21.4%) attended rural schools across Cyprus.

In order to assess the first research question, two stepwise multiple regression analyses were conducted, one for each gender. In these analyses, the criterion variable was the total score of the EAT-26, and the predictor variables were: overall appearance satisfaction, investment in appearance, satisfaction with specific body parts, weight/appearance-related anxiety, internalization of the thin and athletic ideals, pressures from the media, situational body image dysphoria, global self-esteem and body appreciation. The regression equation for males was significant (*F* (5,1057) = 152.21, *p* < 0.001) and indicated that five of the 10 variables were significant predictors of disordered eating in males (see Table 1). More specifically, body appreciation was the most significant variable, which served as a protective factor and accounted for 35.3% of the variance of disordered eating (*R*^2^ = 0.353, *R*^2^*_adjusted_* = 0.353). This was followed by situational body image dysphoria (which contributed another 3.8% to the total variance and served as a risk factor (*R*^2^ = 0.392, *R*^2^*_adjusted_* = 0.391), weight/appearance-related anxiety (adding another 1.6% (*R*^2^ = 0.409, *R*^2^*_adjusted_* = 0.407) and pressures from the media (adding another 0.6%; *R*^2^ = 0.416, *R*^2^*_adjusted_* = 0.413) all of which served as risk factors. Finally, satisfaction with specific parts of the body (0.3%; *R*^2^ = 0.419, *R*^2^*_adjusted_* = 0.416) served as a second, small, but significant, protective factor.

The regression equation for females was also significant (*F* (4,1537) = 323.87, *p* < 0.001) and indicated that four of the 10 variables were significant predictors of disordered eating (see Table 1). More specifically, weight/appearance-related anxiety was the most significant variable, which served as a risk factor and accounted for 33.0% of the variance of disordered eating (*R*^2^ = 0.330, *R*^2^*_adjusted_* = 0.330). This was followed by body appreciation, which contributed another 13.7% to the total variance and served as a protective factor (*R*^2^ = 0.468, *R*^2^*_adjusted_* = 0.467). Furthermore, situational body image dysphoria contributed an additional 3.4% (*R*^2^ = 0.502, *R*^2^*_adjusted_* = 0.501) and thin ideal internalization added another 1.0%; (*R*^2^ = 0.512, *R*^2^*_adjusted_* = 0.511) both, serving as significant risk factors.

These results provide partial support for the first research question. Firstly, body appreciation served as a significant protective factor for both males and females, as predicted, however, global self-esteem did not. Secondly, the results showed some differences in risk factors for males and females, as predicted—however, some similarities were also found. The internalization of the thin ideal was a significant risk factor for females only, and pressures from the media served as a significant risk factor for males only. Additionally, satisfaction with specific parts of the body served as a protective factor for males, but not for females. The athletic ideal internalization did not serve as a risk factor for disordered eating. Situational body image dysphoria and weight/appearance-related anxiety, on the other hand, served as significant risk factors for both males and females.

Three stepwise multiple regression analyses were conducted to test the second research question; one for each BMI category (underweight, normal weight and overweight/obese). In these analyses, the criterion variable was the total score of the EAT-26, and the predictor variables were overall appearance satisfaction, investment in appearance, satisfaction with specific body parts, weight/appearance-related anxiety, internalization of the thin and athletic ideals, pressures from the media, situational body image dysphoria, global self-esteem, and body appreciation.

The regression equation for the underweight category was significant (*F* (5,588) = 91.55, *p* < 0.001) and indicated that five of the 10 variables were significant predictors of disordered eating in underweight individuals (see Table 2). Body appreciation was the most significant variable, which served as a protective factor and accounted for 26.8% of the variance of disordered eating (*R*^2^ = 0.269, *R*^2^*_adjusted_* = 0.268). The next significant predictor was weight/appearance-related anxiety, adding another 12.7% (*R*^2^ = 0.397, *R*^2^_*adjusted*_ = 0.395) followed by situational body image dysphoria, which contributed another 3.1% to the total variance; (*R*^2^ = 0.429, *R*^2^*_adjusted_* = 0.426) and thin ideal internalization, contributing 0.3%; (*R*^2^ = 0.433, *R*^2^*_adjusted_* = 0.429), all which served as significant risk factors. Finally, investment in appearance (0.4%; *R*^2^ = 0.438, *R*^2^*_adjusted_* = 0.433) served as a significant protective factor for disordered eating for individuals in the underweight category.

The regression equation for the normal weight category was also significant (*F* (4,1717) = 425.49, *p* < 0.001) and indicated that four of the 10 variables were significant predictors of disordered eating in normal weight individuals (see Table 2). As with the underweight category, body appreciation was the most significant predictor serving as a protective factor and accounting for 34.0% of the variance of disordered eating (*R*^2^ = 0.340, *R*^2^_*adjusted*_ = 0.340). The next three significant predictors were also identical to the underweight category and served as risk factors: weight/appearance-related anxiety added another 12.6% (*R*^2^ = 0.467, *R*^2^*_adjusted_* = 0.466); situational body image dysphoria, added another 2.3% (*R*^2^ = 0.490, *R*^2^*_adjusted_* = 0.489); and thin ideal internalization contributed (0.8%; *R*^2^ = 0.498, *R*^2^*_adjusted_* = 0.497), all of which served as significant risk factors.

The regression equation for the overweight/obese category was also significant (*F* (4,284) = 57.88, *p* < 0.001), and as with the normal weight category, four of the 10 variables were significant predictors of disordered eating in overweight/obese individuals (see Table 2). As with the underweight and normal weight categories, body appreciation was the most significant predictor, serving as a protective factor and accounting for 25.8% of the variance of disordered eating (*R*^2^ = 0.261, *R*^2^*_adjusted_* = 0.258). Furthermore, and also identical to the underweight and normal weight categories, the risk factors were weight/appearance-related anxiety, which added another 13.3% (*R*^2^ = 0.395, *R*^2^*_adjusted_* = 0.391), situational body image dysphoria, which added another 3.1% (*R*^2^ = 0.429, *R*^2^*_adjusted_* = 0.422), and thin ideal internalization, which contributed a final 1.9% (*R*^2^ = 0.449, *R*^2^*_adjusted_* = 0.441).

The second research question was therefore also partially supported. Body appreciation served as a significant protective factor for all BMI categories, as predicted, however, global self-esteem did not. However, the only difference that was observed between the BMI categories was that in the underweight category, investment in appearance served as a small but significant protective factor for disordered eating. Weight/appearance-related anxiety, situational body image dysphoria and the internalization of the thin ideal, all served as significant risk factors for all BMI categories.

## 4. Discussion

The current study aimed to identify protective and risk factors for the development of eating disorders in a large sample of adolescents, based on their gender and Body Mass Index (BMI). The study is significant to the literature concerning the contribution of psychosocial factors to eating disorders, since it provides additional information about males and eating disorders (who are usually understudied) [29] as well as different BMI groups.

Concerning gender and eating disorders, the results of the study clearly support that weight/appearance-related anxiety served as a significant risk factor for both males and females, and that thin-ideal internalization was a significant risk factor only for females. These findings are in concordance with Keel and Forney’s review [1], which outlines the psychosocial factors contributing to eating disorders, as well as with other previous findings [6,7]. Additionally, the study identifies pressures from the media as a significant risk factor for males, a finding which is also supported by previous research [30]. In addition to these findings, the construct of situational body image dysphoria was found to act as a significant risk factor for the development of eating disorders for both males and females. This indicates that when male and female adolescents experience dysphoria in social settings due to their negative body image, this increases their likelihood of developing disordered eating. This finding is especially significant to the literature, as the construct is largely understudied. The influence of social body image dysphoria should therefore be considered when developing and implementing intervention programs for the prevention of eating disorders.

Interestingly, pressure from the media served as a significant risk factor only for males, contradicting previous literature, which has found pressure from the media to affect both males and females [31]. It is worth noting that, when an exploratory multiple regression analysis was carried out with pressure from the media as the only predictor variable and disordered eating as the criterion variable, pressure from the media did significantly predict 14.8% (*p* < 0.001) of the variance of disordered eating in females. However, when all 10 predictor variables were included in the regression equation, pressure from the media lost its power in predicting disordered eating, since the other variables became stronger predictors. Thus, for the current sample, weight/appearance-related anxiety, situational body image dysphoria, and internalization of the thin ideal were significantly more robust and more significant predictors of disordered eating than pressure from the media. The order of these predictors should also be considered in the development of intervention programs. Specifically, the amount of variance accounted for each variable is hierarchical, suggesting that the first variable reported plays a more significant risk/protective role than the last variable reported. Concerning males, as predicted, pressures from the media served as a significant risk factor, and satisfaction with specific parts of the body served as a protective factor. This finding is also in agreement with previous studies [30]. However, it should be interpreted with caution since, even though it is statistically significant, the amount of variance that pressures from the media added was only 0.06 for males. Additionally, contrary to previous research, the internalization of the athletic ideal did not serve as a predictive factor to disordered eating in males. As was the case with pressure from the media in females, an exploratory multiple regression analysis revealed that when used alone in the equation, the internalization of the athletic ideal was a significant predictor (3% of the variance, *p* < 0.001), however, when the other nine predictors were added, it lost its power. It seems as though the risk factors of weight/appearance-related anxiety, situational body image dysphoria, and pressures from the media were very strong in accounting for the variability of disordered eating. The same is the case for global self-esteem; individually, self-esteem does serve as a protective factor for disordered eating for both genders, but when other predictors are added, it loses its power to the other significant risk and protective factors.

The results of the study also strongly support the importance of the construct of body appreciation as a significant protective factor for both males and females. This conclusion is also in line with previous findings [11]. Body appreciation served as a significant protective factor accounting for the largest part of the variability in males, and the second largest in females, indicating its invaluable contribution to the prevention of eating disorders. Based on these findings, intervention and prevention programs should either already include, or be adjusted to include, exercises that promote the construct of body appreciation. The above findings also imply that body appreciation may be more suitable as a construct to be used as a protective factor in adolescents than global self-esteem.

Concerning BMI and eating disorders, the results of the study clearly support that weight/appearance-related anxiety, situational body image dysphoria, and internalization of the thin ideal all served as significant risk factors for underweight, normal weight and overweight/obese individuals, regardless of BMI. Previous research [32,33] indicates that individuals with a high BMI are more at risk for developing an eating disorder. The current findings further support that the risk factors for developing an eating disorder are identical, regardless of BMI. This should be considered when choosing a targeted population for the designing and implementation of prevention programs. Additionally, body appreciation was the most significant construct, and a protective factor against disordered eating across all BMI categories. As with gender, these results strongly support that the contribution of body appreciation to the prevention of eating disorders is essential. Intervention and prevention programs should either already include, or adjust their material to include exercises that promote body appreciation regardless of the participants’ BMI. Similarly, the above findings also imply that the construct of body appreciation, compared to global self-esteem, seems more suitable to be used as a protective factor in adolescents, regardless of their body weight and height.

A final finding worth considering is that investment in appearance acted as a protective factor for individuals in the underweight category. Even though previous research had found investment in appearance to act as a risk factor [14], the findings were not specific to underweight individuals. It is possible that investment in appearance results in overall satisfaction with appearance, thus decreasing the likelihood of disordered eating in underweight individuals. The same is the case with satisfaction with specific parts of the body that served as a protective factor in males.

### Limitations and Future Research

The findings of the current study should be considered in the context of several limitations. First, the data of the study were collected through the use of self-report questionnaires, in which participants were asked to provide their height and weight. Past studies have found that both males and females tend to underestimate their weight and overestimate their height [12]. Therefore, the findings concerning BMI are subject to biases due to participants either over or underestimating these factors, thus possibly affecting a significant variable used in this study (BMI). Second, the findings’ generalizability should be carried out with caution and considering the following factors regarding the sample (a) the age range; and (b) geography. More specifically, the current sample comprised adolescents; therefore, the risk and protective factors may differ for other age groups. Additionally, certain factors related to the geography of Cyprus, which have been found to play a role in body image and eating disorders, may not apply to other cultural groups, leading to differences in risk and protective factors between cultures.

Several ideas concerning the direction of future research arise from the current study. As already mentioned in the introduction, body appreciation and self-compassion have served as protective factors for disordered eating in the past. In the current study, body appreciation was a clear and robust protective factor, even better than global self-esteem. Therefore, future studies could include self-compassion in their research to assess the robustness of self-compassion as compared to body appreciation. Moreover, future research may use alternative statistical methodology, such as structural equation modelling, to assess the variables’ structural relationships and directions. Additionally, future studies may wish to replicate the current findings using different methodologies (e.g., mixed or qualitative research) or using anthropometric methods of calculating BMI. Future research may also focus further on the construct of situational body image dysphoria and attempt to identify its role more specifically as a risk factor for eating disorders. The protective roles of investment in appearance in the underweight population and satisfaction with specific parts of the body for males should also be further assessed. Finally, future studies may wish to identify/compare risk and protective factors across different age groups and different cultures/populations.

## 5. Conclusions

The current study aimed to identify risk and protective factors in the development of eating disorders in adolescents and determine whether or not these factors differ between genders and Body Mass Index (BMI) categories. The findings emphasized the role of weight/appearance-related anxiety and situational body image dysphoria as the most significant risk factors in the development of eating disorders in both male and female adolescents. Additional risk factors were also identified; however, these differed between males and females. Pressures from the media posed a significant risk for the development of eating disorders in males, but not in females. Additionally, the internalization of the thin-ideal posed a significant risk factor for the development of eating disorders in females. However, the internalization of the athletic-ideal did not pose a significant risk factor for males. Moreover, BMI categories seem to have very similar risk and protective factors.

This study also highlighted the significance of body appreciation as a vital protective factor against the development of eating disorders for both male and female adolescents and across all BMI groups. Body appreciation was found to be a more potent protective factor than global self-esteem. This finding is particularly significant in informing intervention and prevention programs, which would benefit from the inclusion of exercises that promote the construct of body appreciation. Finally, situational body image dysphoria in male and female adolescents should be taken into consideration in intervention and prevention programs as well.

## Figures and Tables

**Table 1:** Summary of multiple regression of males and females predicting disordered eating.

Independent Predictor Variables	B	S.E.	*β*	t	Sig.	Adjusted R^2^
MALES (*n* = 1063)						
Body Appreciation	−0.668	0.032	−0.516	−20.99	0.000 ***	0.353
SIBID-S Body Image Dysphoria	1.058	0.265	0.110	3.99	0.000 ***	0.391
MBSRQ Overweight Preoccupation (Weight/Appearance-Related Anxiety)	1.009	0.272	0.104	3.71	0.000 ***	0.407
SATAQ-3 Pressures from Media	0.154	0.047	0.092	3.28	0.001 **	0.413
MBSRQ Body Areas Satisfaction	−0.668	0.282	−0.060	−2.37	0.018 *	0.416
FEMALES (*n* = 1542)						
MBSRQ Overweight Preoccupation (Weight/Appearance-Related Anxiety)	3.731	0.252	0.327	14.82	0.000 ***	0.330
Body Appreciation	−0.566	0.030	−0.351	−18.63	0.000 ***	0.467
SIBID-S Body Image Dysphoria	2.079	0.260	0.177	8.00	0.000 ***	0.501
SATAQ-3 Thin Ideal Internalization	0.162	0.029	0.121	5.48	0.000 ***	0.511

Note.—Total *N* = 2605. * *p* < 0.05, ** *p* < 0.01, *** *p* < 0.001.

**Table 2:** Summary of multiple regression of BMI categories predicting disordered eating.

Independent Predictor Variables	B	S.E.	*β*	t	Sig.	Adjusted R^2^
UNDERWEIGHT (*n* = 594)						
Body Appreciation	−0.562	0.047	−0.392	−12.07	0.000 ***	0.268
MBSRQ Overweight Preoccupation (Weight/Appearance-Related Anxiety)	2.764	0.401	0.266	6.89	0.000 ***	0.395
SIBID-S Body Image Dysphoria	2.093	0.417	0.183	5.02	0.000 ***	0.426
SATAQ-3 Thin Ideal Internalization	0.121	0.047	0.099	2.57	0.011 *	0.429
MBSRQ Appearance Orientation (Investment in Appearance)	−1.096	0.502	−0.073	−2.18	0.029 *	0.433
NORMAL WEIGHT (n = 1722)						
Body Appreciation	−0.624	0.028	−0.416	−22.42	0.000 ***	0.340
MBSRQ Overweight Preoccupation (Weight/Appearance-Related Anxiety)	2.854	0.228	0.265	12.50	0.000 ***	0.466
SIBID-S Body Image Dysphoria	1.615	0.233	0.145	6.92	0.000 ***	0.489
SATAQ-3 Thin Ideal Internalization	0.147	0.028	0.110	5.27	0.000 ***	0.497
OVERWEIGHT/OBESE *(n* = 289)						
Body Appreciation	−0.600	0.071	−0.388	−8.47	0.000 ***	0.258
SIBID-S Body Image Dysphoria	2.260	0.494	0.239	4.57	0.000 ***	0.422
MBSRQ Overweight Preoccupation (Weight/Appearance-Related Anxiety)	1.920	0.560	0.173	3.43	0.001 **	0.391
SATAQ-3 Thin Ideal Internalization	0.220	0.068	0.163	3.26	0.001 **	0.441

Note.—Total *N* = 2605. * *p* < 0.05, ** *p* < 0.01, *** *p* < 0.001.
